# Use of *Clostridium perfringens* Enterotoxin and the Enterotoxin Receptor-Binding Domain (C-CPE) for Cancer Treatment: Opportunities and Challenges

**DOI:** 10.1155/2012/981626

**Published:** 2011-09-15

**Authors:** Zhijian Gao, Bruce A. McClane

**Affiliations:** ^1^Division of Gynecologic Oncology, Department of Obstetrics, Gynecology, and Reproductive Biology, Brigham and Women's Hospital, Harvard Medical School, Boston, MA 02115, USA; ^2^Department of Microbiology and Molecular Genetics, University of Pittsburgh School of Medicine, 420 Bridgeside Point II Building, 450 Technology Drive, Pittsburgh, PA 15219, USA

## Abstract

*Clostridium perfringens* enterotoxin (CPE) causes the symptoms associated with several common gastrointestinal diseases. CPE is a 35 kDa polypeptide consisting of three structured domains, that is, C-terminal domain I (responsible for receptor binding), domain II (responsible for oligomerization and membrane insertion), and domain III (which may participate in physical changes when the CPE protein inserts into membranes). Native CPE binds to claudin receptors, which are components of the tight junction. The bound toxin then assembles into a hexameric prepore on the membrane surface, prior to the insertion of this oligomer into membranes to form an active pore. The toxin is especially lethal for cells expressing large amounts of claudin-3 or -4, which includes many cancer cells. Initial studies suggest that native CPE has potential usefulness for treating several cancers where claudin CPE receptors are overexpressed. However, some challenges with immunogenicity, toxicity, and (possibly) the development of resistance may need to be overcome. An alternative approach now being explored is to utilize C-CPE, which corresponds approximately to receptor binding domain I, to enhance paracellular permeability and delivery of chemotherapeutic agents against cancer cells. Alternatively, C-CPE fusion proteins may prove superior to use of native CPE for cancer treatment. Finally, C-CPE may have application for other medical treatments, including vaccination or increasing drug absorption. The coming years should witness increasing exploitation of this otherwise formidable toxin.

## 1. Introduction to *Clostridium perfringens* Enterotoxin (CPE)

*Clostridium perfringens* is a major pathogen of humans and livestock [1]. This Gram-positive anaerobe causes both histotoxic infections, such as gas gangrene (clostridial myonecrosis), and enteric infections, such as human food poisoning. As typical amongst pathogenic clostridial spp., the virulence of *C. perfringens* is largely attributable to its ability to produce an arsenal of potent protein toxins. Production of four of these toxins (alpha, beta, epsilon, and iota) is used to classify *C. perfringens* strains into one of five types (A–E). Less than 5% of all *C. perfringens* type A isolates produce another toxin named *C. perfringens* enterotoxin (CPE) that is biomedically important, although not used in the toxin typing classification system [2]. After a brief introduction to this unique toxin, promising efforts to utilize CPE, or its derivatives, for cancer therapy will be described

### 1.1. The Role of CPE in Natural Disease

CPE-producing *C. perfringens* type A strains cause the second most common bacterial food poisoning in the USA, as well as many cases of human nonfoodborne gastrointestinal diseases, such as antibiotic-associated diarrhea [2]. The food poisoning develops when foods contaminated with large numbers of CPE-producing type A strains are ingested; the ingested bacteria then briefly grow in the small intestine before committing to sporulation. It is during this *in vivo* sporulation that the enterotoxin is produced. Molecular Koch's postulates analyses have demonstrated that production of CPE is essential for CPE-positive type A human food poisoning or nonfoodborne gastrointestinal disease isolates to cause gastrointestinal effects in animal models [3].

Most, but not all, *C. perfringens* type A food poisoning strains carry their enterotoxin gene (*cpe*) on the chromosome [2]. In contrast, the* cpe *gene of type A nonfoodborne human disease strains is located on large (~70–75 kb) plasmids [4]. Amongst *cpe*-positive type A strains there are two major families of *cpe* plasmids [5]. These enterotoxin plasmids can be conjugative [6], presumably due to the presence of a Tn*916*-like region named *tcp* that has been experimentally shown to mediate transfer of other *C. perfringens* conjugative plasmids [7]. 

Expression of the *cpe* gene is regulated by sporulation-associated alternative sigma factors [8, 9]. Specifically, one alternative sigma factor (SigF) controls expression of two other alternative sigma factors (SigK and SigE), which then direct transcription of *cpe* mRNA from several SigK- or SigE-dependent promoters located upstream of the *cpe* ORF. Exceptionally large amounts of CPE can be produced during sporulation; for example, CPE can represent 20% of total protein in some sporulating CPE-positive *C. perfringens *type A strains [2]. 

Once produced, CPE is not immediately secreted [2]. Instead it accumulates in the cytoplasm of the mother cell until the completion of sporulation. When the mother cell then lyses to release the mature spore, CPE is released into the intestinal lumen, where it binds and acts as described in the following section. 

The *in vivo* outcome of natural CPE action during gastrointestinal disease is desquamation of the intestinal epithelium, intestinal necrosis, and the accumulation of luminal fluid [1]. These effects account for the natural gastrointestinal symptoms of CPE-associated disease, which most commonly include diarrhea and abdominal cramps. Typically, people are sickened with *C. perfringens* type A food poisoning for 12–24 hours and then recover. However, this illness can be fatal in the elderly or in people suffering from medication-induced constipation [10].

### 1.2. The Cellular Action of CPE

**Figure 1:**
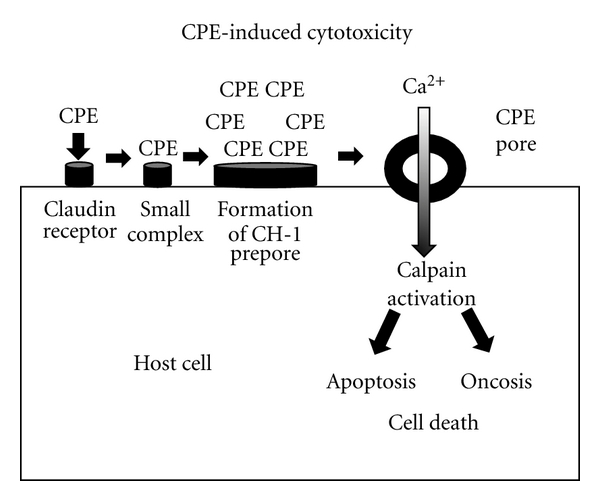
Model for CPE-induced cytotoxicity. CPE first binds to claudin receptors to form an ~90 kDa small complex. Six small complexes are then thought to oligomerize on the membrane surface to form a CH-1 prepore. The prepore then inserts into membranes to form the active pore. This results in entry of calcium into cells, which activates calpain. When a high CPE dose is used, there is substantial entry of calcium into cells, causing a strong calpain activation; this results in cell death by oncosis. Lower CPE doses cause more limited calcium influx and thus a milder calpain activation; these cells die by classical caspase 3-mediated apoptosis.

As shown in Figure 1, the current model of CPE action begins with binding of this toxin to claudin receptors (described in detail below). This binding results in formation of a small (~90 kDa) SDS-sensitive complex. Besides CPE, the small complex also contains [11] both claudin receptors and claudins incapable of binding CPE (i.e., nonreceptor claudins). Presumably the presence of nonreceptor claudins in small complex is attributable to claudin: claudin interactions. Six small complexes are then thought to oligomerize into an SDS-resistant large complex named CPE hexamer-1, or CH-1. This hypothesis is based upon results of heteromer gel shift analyses, which identified the presence of six CPE molecules in each CH-1 complex [11]. CH-1 is ~450 kDa in size and contains, in addition to six CPE molecules, both receptor and nonreceptor claudins [11]. CH-1 initially assembles as a prepore on the membrane surface; however, at 37°C this prepore then rapidly inserts into membranes to form an active pore [12].

 Formation of the CH-1 pore results in calcium influx, which (via calpain activation) leads to cell death [13, 14]. At moderate CPE doses, where modest CPE pore formation allows only limited calcium influx, CPE-treated cells die from a classical caspase 3-mediated apoptosis. At higher CPE doses, where large amounts of CPE pore formation results in a massive calcium influx, cells die from oncosis. 

CPE pore formation also leads to morphologic damage that exposes the basolateral surface of cells. This allows formation of a second bigger (~650 kDa) large complex named CH-2 [15]. In addition to six copies of CPE and both receptor and nonreceptor claudins, CH-2 also contains another tight junction protein named occludin [11, 15]. Formation of CH-2 leads to internalization of occludin into the cytoplasm [11]; claudins are also internalized inside native CPE-treated cells although it is not clear if this is due to CH-1 formation, CH-2 formation, or to formation of both complexes. These effects likely help to explain the observed ability of native CPE to disrupt tight junctions [16].

## 2. The CPE Structure/Function Relationship

### 2.1. Cytotoxicity Domains of CPE

**Figure 2:**
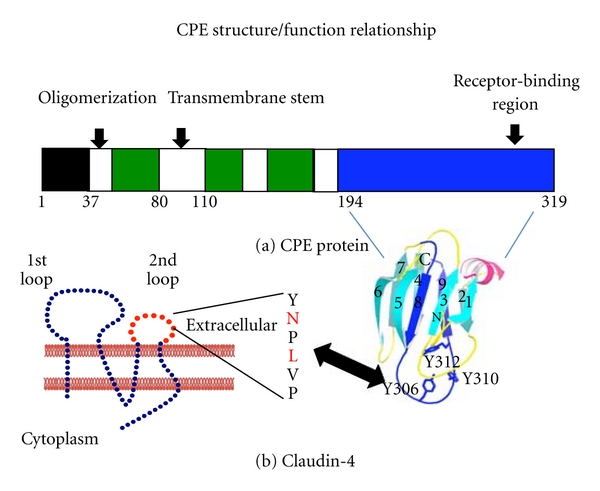
CPE structure versus function relationship. Panel A shows the functional regions of CPE, including the unstructured N-terminal sequences comprising amino acids 1–37 (black box), the domain II sequences mediating oligomerization and membrane insertion (white boxes), the domain III sequences that may participate when CPE inserts into membranes (green boxes), and the domain I sequences that mediate CPE receptor binding (blue box). Shown below the drawing is the structure of domain I (used with permission from [17]), including three tyrosine residues that interact with claudin receptors. Panel B shows the predicted structure of claudins. The amino acids in the turn region of extracellular loop 2 are shown to the right of claudin, with the two residues (N and, to a lesser extent, L) important for CPE binding highlighted in red.

CPE consists of a 319 amino acid polypeptide (Mr 35,317) with a unique primary sequence [2]. The CPE structure/function relationship has been extensively analyzed by combined genetic, biochemical, and structural biology approaches (Figure 2). As this review was being prepared, the structure of the native CPE has just been reported [18]. This structure revealed that CPE is a three-domain protein, reminiscent of several other pore-forming toxins.

The N-terminal 37 amino acids of native CPE lack a definable structure and are not necessary for toxicity [2]. In fact, removing these amino acids by chymotrypsin, as may occur in the intestines during disease, produces a 2–3-fold more potent toxin [2]. This proteolytic activation occurs because removal of the N-terminal CPE sequences exposes the CPE region between amino acids 47 to 51, which reside in domain II, to promote CH-1 formation. Thus, this CPE region apparently functions as a latch that facilitates CPE oligomerization. Site-directed mutagenesis studies showed that particularly important domain II residues for CH-1 formation are (i) the Asp at CPE residue 48 and (ii) the Ile at CPE residue 51 [19]. 

CPE domain II also contains a region spanning from residues 81 to 106 that appears to be a transmembrane stem [12]. Removal of these residues produces a CPE variant that can form CH-1 but is unable to kill cells or form pores. This effect is consistent with CPE residues 81 to 106 mediating the insertion of this transmembrane stem into membranes. Membrane insertion of all six transmembrane stems, from the six CPE proteins present in CH-1, results in *β*-barrel pore formation. 

Domain III may undergo structural changes during the prepore to pore transition that facilitate insertion of the CPE transmembrane stem into lipid bilayers, facilitating pore formation [18]. 

### 2.2. Receptor-Binding Domain of CPE

Over 20 years ago it was shown that a recombinant CPE fragment corresponding to the C-terminal half of the native toxin retains full receptor-binding activity [2]. However, (as expected from the preceding section) this C-terminal CPE fragment named C-CPE was nontoxic since it lacks the N-terminal regions necessary for CH-1 formation and insertion of CH-1 into membranes to form a pore. Deletion mutagenesis and synthetic peptide approaches later localized [2] most CPE receptor-binding activity to the 30 C-terminal amino acids (Figure 2). More recently, site-directed mutagenesis studies demonstrated that three Tyr residues, located at positions 306, 310, and 312 of the native toxin protein are important for receptor binding [21]. 

C-CPE (residues 194 to 319 of the native toxin) approximately corresponds to domain I of the native CPE protein [17, 18]. Domain I consists of a nine beta strand sandwich that shares structural similarity with the receptor binding domains of some other bacterial toxins, including the large Cry family of *Bacillus thuringiensis* toxins [17]. By correlating this C-CPE structure with the previous binding-activity mapping studies described above, it is apparent that the receptor-binding site of CPE resides on a large loop located between beta strands 8 and 9. 

## 3. Claudins as CPE Receptors

### 3.1. Introduction to the Claudins

Mammalian tight junctions act as both fences and gates, that is, they represent important barriers for an epithelium and also regulate paracellular permeability [22]. Studies conducted over the past 15 years have determined that the tight junction is comprised of several proteins, the most important of which are the claudins [22]. The claudins are a 24-member family of ~20–25 kDa proteins that are predicted to consist of four transmembrane domains, two extracellular loops (ECL-1 and ECL-2), and a cytoplasmic tail that can mediate signaling cascades. Claudins polymerize into strands that comprise much of the tight junction. The distribution of individual claudins varies amongst different tissues. As described in detail later, claudins are also overexpressed in many cancers.

### 3.2. Evidence that CPE Binds to Claudin Receptors

#### 3.2.1. Fibroblast Transfectant Studies

In 1997, Katahira et al. reported that when fibroblasts, which are naturally CPE- resistant, were transfected to express an ~22 kDa Vero cell protein, they gained CPE sensitivity [23]. The Vero cell protein expressed by these transfectants had properties of a CPE receptor since the fibroblast transfectants acquired the ability to bind significant levels of the toxin and to form high molecular weight complexes that are now recognized as CH-1. This Vero cell CPE receptor protein was later identified as claudin-4. It was also determined [24] that, at physiologic concentrations, the enterotoxin can bind to transfected fibroblasts expressing claudin-3, -4, -6, -8, or -14. However, no CPE binding was detected to transfectants expressing claudins-1, -2, -5, or -10. 

#### 3.2.2. Studies with Naturally CPE-Sensitive Enterocyte-Like Cells

A more recent study by Robertson et al. demonstrated that CPE also interacts with claudins in naturally CPE-sensitive Caco-2 cells, which are a human enterocyte-like cell line [11]. Using coimmunoprecipitation and electroelution approaches, this study showed both the CPE small complex and CH-1 large complex formed in Caco-2 cells can contain, in addition to CPE, receptor claudins-3 and -4, along with the nonreceptor claudin-1. In addition to CPE, receptor claudins, and nonreceptor claudins, the CH-2 complex formed in Caco-2 cells also contains occludin [15]. The stoichiometry of claudins in CH-1 and CH-2, or occludin in CH-2, has not yet been determined. 

### 3.3. Mapping of the CPE Binding Site in Claudin Receptors

The structure of a claudin has not been solved at the time when this review is being prepared. However, as mentioned earlier (and depicted in Figure 2), claudins are predicted to possess two extracellular loops named ECL-1 and ECL-2. An early study using claudin chimeras consisting of the N-terminal half of CPE receptor claudin-4 fused with the C-terminal half of CPE nonreceptor claudin-1 suggested that CPE interacts with the putative ECL-2 region [24]. This hypothesis was rigorously confirmed by a recent study [25] using more specific chimeric claudins, which showed that substituting only the predicted ECL-2 sequence of claudin-4 into a claudin-1 backbone is sufficient to produce fibroblast transfectants that are very sensitive to CPE. The reverse was also true, that is, transfectants expressing a chimeric claudin, where only the claudin-1 ECL-2 had been specifically substituted into the claudin-4 backbone, were CPE-insensitive. 

Several recent studies (reviewed in [26]) have focused on understanding the specific ECL-2 residues of claudin receptors that mediate CPE binding. These studies have utilized a variety of approaches including arrays of immobilized ECL-2 synthetic peptides, solubilized claudins, or transfectants expressing claudin mutant. Results from these studies suggested that ECL-2 possesses a helix-turn-helix motif that interacts with CPE. An Asn residue in the middle of this turn appears to be important for CPE binding; some evidence suggests that a Leu residue located two residues from this Asn may also participate in the binding of this toxin. 

## 4. CPE and Cancer Treatment

### 4.1. Introduction

Since approximately 85% of malignant tumors are derived from epithelial cells, an epithelium-targeted therapeutic strategy has been the focus of cancer translational research. As mentioned earlier, claudins are the major components of paracellular tight junctions (TJs), distribute at the most apical junctions between epithelial cells, and play an essential role in the control of paracellular transport. Furthermore, Claudin-3 and -4 have been identified as the specific receptors for CPE, which is of potential therapeutic significance since these two claudins are abundantly expressed in ovarian, breast, uterine, and pancreatic cancers [27]. While CPE can trigger lysis of epithelial cells by binding to claudin-3 and claudin-4, with resultant initiation of massive permeability changes, osmotic cell ballooning, and cytolysis within 5–15 min (see previous sections of this review), cells lacking expression of the CPE receptors are completely unaffected by this enterotoxin [19]. These observations have raised the possibility that CPE may be an innovative claudin-targeted therapy for malignant tumors. In fact, efforts have been made to use CPE in the treatment of claudin-overexpressing cancers during the past few years.

### 4.2. CPE Treatment and Ovarian Cancer

Ovarian cancer remains the most lethal gynecologic malignancy in the United States. Approximately 90% of patients with advanced-stage ovarian cancer develop recurrence and inevitably die from the development of chemotherapy resistance. Therefore, the discovery of novel and effective therapy is of immediate clinical importance. Previous studies have demonstrated that claudin-3 and -4 were among the six most differentially upregulated genes in ovarian cancer cells, but their expression is undetectable in normal ovaries. Of particular note, chemotherapy-resistant/recurrent ovarian cancers express claudin-3 and -4 at significantly higher levels than chemotherapy-sensitive cancers [28]. 

These overexpressed claudins may represent promising targets for the use of CPE as a tumor-targeting therapy against this aggressive disease. Indeed, Santin and colleagues successfully used CPE to treat an animal model of chemotherapy-resistant human ovarian cancer [29]. They found that all ovarian cancer cells, regardless of their resistance to chemotherapeutic agents, showed a dose-dependent cytotoxic response and died rapidly after 24 hours of exposure to CPE. Furthermore, in this animal model employing chemotherapy-resistant human ovarian cancer xenografts, multiple intraperitoneal (i.p.) sublethal doses of CPE ranging from 5 to 8.5 *μ*g/mL significantly inhibited tumor growth and extended the survival of mice harboring a large tumor burden of chemotherapy-resistant ovarian cancer. The application was well tolerated throughout the treatment period. Therefore, CPE-based therapy may have potential as a novel treatment for chemotherapy-resistant ovarian cancer.

### 4.3. CPE Treatment and Breast Cancer

Breast cancer is one of the most common malignancies worldwide. Despite tamoxifen and aromatase inhibitor having significantly improved long-term survival of patients diagnosed at the early stages, advanced breast cancer and metastatic breast cancer are still incurable diseases. Claudin-3 and claudin-4 are overexpressed in most primary breast carcinomas and breast cancer brain metastases but undetectable in normal breast epithelial cells. In 2004, Kominsky et al. reported [30] for the first time that intratumoral CPE treatment of T47D human breast cancer cell xenografts resulted in significant tumor suppression and necrosis in SCID mice without any side effects. However, i.p. administration of the same dose of CPE was toxic and had no effect on tumor volume. CPE also damaged breast cancer cell lines in a claudin-dependent manner but did not affect cell lines lacking claudin-3 and -4 [30]. 

**Figure 3:**
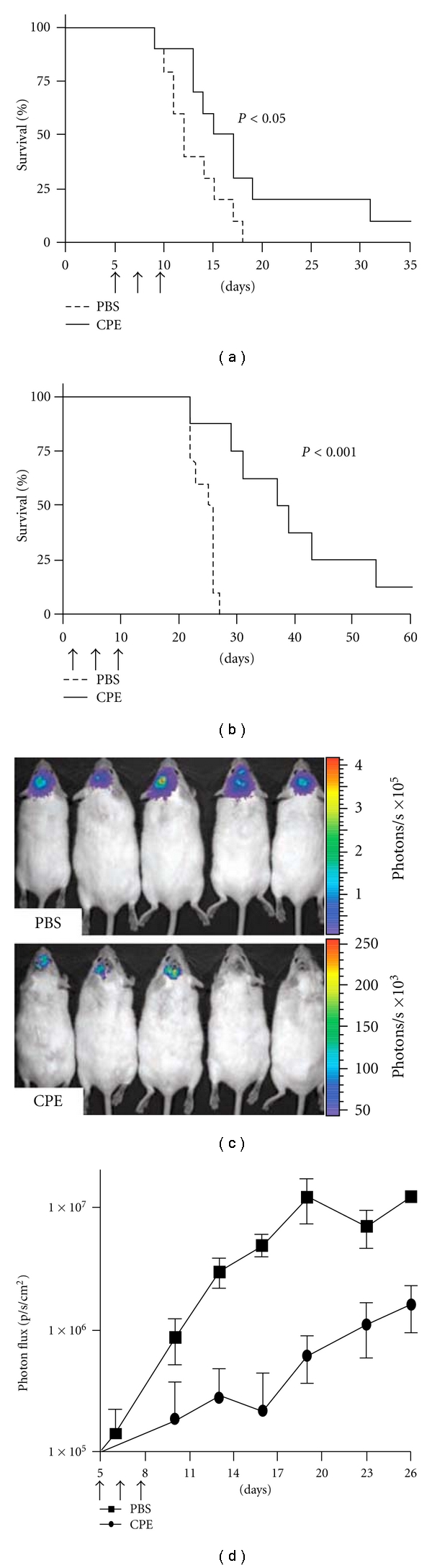
Efficacy of CPE in the treatment of breast cancer brain metastasis. (a, b) Brain tumors were established in mice using the human breast cancer cell line MDA-MB-468 and the murine breast cancer cell line NT2.5-luc. Tumors were treated by intracranial administration of 0.5 *μ*g CPE versus PBS on days 5, 7, and 9. (c, d) For the NT2.5-luc brain tumor model, noninvasive bioluminescent imaging was done twice per week beginning on day 4. Bioluminescent images from five representative mice are shown for each experimental group at day 19 (c). Photon flux was measured over the indicated time course as an indication of tumor growth (d). Differences in survival between experimental groups were analyzed using the log-rank test. Reproduced with permission from [20].

In later studies [20], those investigators found that intracranial administration of CPE retarded tumor growth and increased survival in two murine models of breast cancer brain metastasis without any apparent systematic or CNS toxicity (Figure 3). These two studies suggest that the local administration of CPE may be useful in the treatment of breast cancer.

Overexpression of claudin-3 and -4 has been found in uterine serous papillary carcinoma (USPC) and correlates with a more aggressive phenotype and a worse prognosis. A study by Santin et al. has demonstrated that CPE effectively and specifically triggers cytolysis of primary and metastatic USPC cell lines in a dose-dependent manner whereas normal cells lacking claudin-3 and -4 are unaffected by CPE treatment [31]. In particular, multiple intratumoral injections of CPE in large subcutaneous USPC xenografts led to tumor necrosis and even tumor disappearance in 100% of animals. Similarly, i.p. injection of sublethal doses of CPE significantly suppressed tumor growth and extended survival of animals harboring chemotherapy-resistant intra-abdominal USPC. The local/regional administrations of CPE were well tolerated without observable adverse events in animals.

### 4.4. CPE Treatment of Pancreatic and Prostate Cancers

CPE has also been used to treat pancreatic and prostate cancers in nude mice or *in vitro *by different research groups. Michl et al. reported that intratumoral injections of CPE in pancreatic cancer xenografts resulted in apparent tumor suppression and necrosis in mice, and that CPE treatment also caused an acute dose-dependent cytotoxic effect in pancreatic cancer cells *in vitro *[32]. In prostate cancer, Long et al. showed that the prostate cancer metastatic cells from the bone marrow were sensitive to CPE-mediated cytolysis* in vitro* [33]. These two preclinical studies suggest that CPE may have potential as a novel therapy for primary and metastatic malignancies expressing claudin-3 and -4.

### 4.5. Challenges of Using Native CPE for Cancer Therapy

Although the specificity and rapidity of CPE-mediated cytolysis may increase anticancer efficacy and reduce opportunity for the development of drug resistance, its clinical application encounters some challenges. Since claudin-3 and -4 are expressed in some normal tissues such as prostate, lung, and the gastrointestinal tract, systemic toxicity is an important concern for CPE therapy. In this context, many investigators have chosen the local administration of native CPE to treat cancers. This approach, however, does not appear to be a good option for some metastatic cancers such as lung metastasis or multiple metastases. Moreover, some adverse events have sometimes been observed even following the local administration of CPE during several studies [30, 34]. Furthermore, CPE is recognized as a virulence factor responsible for the pathophysiological responses associated with a common food poisoning, proinflammatory cytokine response and other human diseases [34]. Another potential problem would include possible immune responses against CPE. Since CPE-associated foodborne illness is so common, many people already have serum antibodies against this toxin. It is unclear whether these serum CPE antibodies include neutralizing antibodies but CPE does contain at least one neutralizing epitope, present in the CPE binding domain [2]. Other challenges include determining optimal dosage and regimen, development of drug resistance, and long-term safety concerns. Therefore, further detailed studies will be required to resolve these issues.

## 5. C-CPE and Cancer Treatment

Although the clinical application of CPE is limited by its potentially significant toxic side effects, the C-terminal binding domain of CPE (C-CPE) overcomes this drawback of CPE and has recently emerged as a promising cancer therapeutic agent due to its unique properties. For example, C-CPE can disrupt the paracellular TJ barrier by binding to claudin-3 and -4 in the epithelia and thus improve drug delivery in a noncytotoxic fashion. It also has a smaller molecular size that might provide less immunogenicity than CPE. 

The paracellular TJs are the primary barrier to the transport of solutes from the apical surface to the core of cells. Agent uptake via the paracellular pathway in the epithelia is considered an attractive route for the absorption of chemotherapies. Given that claudin-3 and -4 are overexpressed in several cancers and are major components of cell TJs, their downregulation by C-CPE may prove a novel strategy for enhancing conventional chemotherapy delivery into claudin-positive cancer cells [36]. 

**Figure 4:**
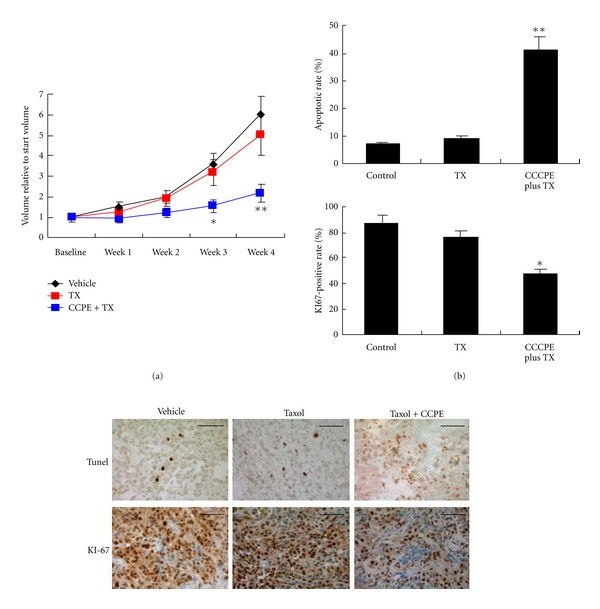
Combination therapy of C-CPE and taxol attenuates EOC xenograft growth *in vivo*. Female SCID mice were inoculated s.c. with 5 × 10^6^ SKOV-3 cells. Four weeks later, the mice harboring large tumor burden were intraperitoneally administered with taxol alone (20 mg/kg), taxol combined with C-CPE (0.1 mg/kg), or vehicle (PBS) twice a week for 4 weeks. (a) Growth curves of tumors were presented as the mean volume normalized to the start volume. *The combination of C-CPE and taxol led to a significant tumor suppression compared with vehicle or taxol alone (*P* < 0.05). (b) After 4 weeks of treatment, immunostaining of Ki67 and TUNEL was performed to evaluate cell proliferation and apoptosis in EOC xenografts. *P < 0.05; **P < 0.001. Reproduced with permission from [35].

Our group recently investigated the efficacy of a combination therapy using a chemotherapeutic agent with C-CPE as compared to the single-agent chemotherapeutic agent [35]. Using three-dimensional and monolayer culture models and a xenograft mouse model of human EOC cells, we found that C-CPE enhanced the chemosensitivities of EOC cell lines to Taxol or Carboplatin at low concentrations in a claudin-dependent fashion. Moreover, repeated i.p. administration of C-CPE in combination with Taxol significantly suppressed large tumor burdens by about 59% compared with control or Taxol alone and showed no apparent toxic drawback of CPE as encountered in previous studies (Figure 4). Our study suggests that, at relatively low concentrations, C-CPE may enhance the sensitivity of EOC cells and other claudin-sensitive tumor cells to conventional chemotherapy and thus alleviate systemic side effects of the agents.

Indeed, C-CPE could be useful not only as an anticancer drug enhancer but also as a carrier to deliver a variety of toxins leading to a spectrum of new anticancer drugs of high selectivity. TNF-*α* has been demonstrated to be an attractive antitumor agent in a variety of animal models but clinical application has been limited due to its failure to concentrate at the site of tumors and the development of severe side effects. To solve the problem, Yuan et al. engineered a C-CPE-TNF fusion toxin that was >6.7-fold more cytotoxic than free TNF to ovarian cancer cells expressing claudin-3 and -4; whereas the TNF component in the fusion was 5-fold less potent than free TNF, suggesting that C-CPE-TNF fusion may prevent or decrease the systemic side effects caused by TNF [37]. Besides ovarian cancer, Saeki et al. fused C-CPE to the protein synthesis inhibitory factor (PSIF) derived from *Pseudomonas aeruginosa *exotoxin A for breast cancer that expresses high levels of claudin-3 and -4 [38]. The C-CPE-PSIF fusion selectively damaged claudin-expressing breast cancer cell lines, and repeated intratumoral injections significantly suppressed tumor size by 36% without causing apparent side effects in mice [38]. These two studies indicate that C-CPE may be a useful carrier for delivering toxins to claudin-sensitive malignancies with higher anticancer potency and less systemic side effects. 

The advantages of this claudin-targeted C-CPE-based cancer therapy are significant: tumor-targeted therapy, less immune response, and an improved therapeutic potency not achieved with previous treatments. There remain challenges to be overcome, however, before we can see any major medical advances in treating cancer with C-CPE: (1) determining safe dosages and schedules, (2) choosing optimal administration routes, (3) avoiding potential immunogenicity, and (4) improving effectiveness for preventing and/or treating cancer metastases. Therefore, further studies and clinical trials are required to determine whether C-CPE could be developed as a claudin-targeted novel therapeutic agent for the treatment of cancer.

## 6. Use of C-CPE for Other Medical Applications

In addition to cancer treatment, C-CPE has been utilized to treat other medical conditions. For example, with the intention of developing a potent mucosal vaccination approach, a nasal vaccine of C-CPE-fused antigen has been prepared and applied in mice without mucosal injury and side effects [39]. In addition, the ability of C-CPE to enhance paracellular permeability has been exploited by using this protein to increase drug absorption from the intestines [40]. One potential issue regarding the use of C-CPE for increasing intestinal drug absorption could be possible gastrointestinal side effects such as diarrhea, due to increased paracellular permeability. However, C-CPE does not increase luminal fluid levels in rabbit ileal loops, possibly arguing against this concern [41].
